# Investigating the Effects of Embodiment on Emotional Categorization of Faces and Words in Children and Adults

**DOI:** 10.3389/fpsyg.2019.02871

**Published:** 2020-01-15

**Authors:** Michael Vesker, Daniela Bahn, Christina Kauschke, Mareike Neumann, Cecilia Sweitzer, Gudrun Schwarzer

**Affiliations:** ^1^Department of Developmental Psychology, University of Giessen, Giessen, Germany; ^2^Department of Clinical Linguistics, University of Marburg, Marburg, Germany

**Keywords:** face perception, word perception, emotion perception, embodiment, facial feedback hypothesis

## Abstract

The facial feedback hypothesis (FFH) indicates that besides being involved in the production of facial expressions, the musculature of the face also influences one’s perception of emotional stimuli. Recently, this effect has been the focus of increased scrutiny as efforts to replicate a key study with adult participants supporting this hypothesis, using the so-called “pen-in-the-mouth” task, have not been successful at several labs. Our series of experiments attempted to investigate whether the assumed embodiment effect can be reproduced in a simplified emotional categorization task for emotional faces and words. We also wanted to test whether the embodiment effect can be detected in children because it is assumed that their bodily processes are especially closely linked with their sensory and cognitive processes. Our experiments involved child and adult participants categorizing faces and words as positive or negative as quickly as possible, while inducing a positive or negative facial or bodily state (holding a straw in the mouth such that a smile or a frown was generated, or creating a positive or negative body posture). The positive or negative facial and bodily states could therefore be either congruent or incongruent with the valence of the target face and word stimuli. Our results did not show any significant differences between the congruent and incongruent conditions in either children or adults. This suggests that embodiment effects either do not significantly impact valence-based categorization or are not strong enough to be detected by our approach considering the sample size in the present study.

## Introduction

The facial feedback hypothesis (FFH) posits that the musculature related to the formation of emotional facial expressions also influences the perception of emotional stimuli. Although ideas regarding the influence of the state of the body on the perception of emotional stimuli were proposed by Darwin (1872) over a century ago in “The Expressions of Emotions in Man and Animals,” the FFH as we understand it today is often credited to Silvan Tomkins (1962) and has gathered a great deal of support by the early 1980s, 1990s, and early 2000s (Buck, 1980; Laird, 1984; Niedenthal et al., 2005). Perhaps most notably, the seminal 1988 paper by Strack, Martin, and Stepper demonstrated the FFH using the so-called “pen-in-the-mouth” task, which allowed for the facilitation or inhibition of smiling without asking participants to pose expressions or contract particular muscles, allowing for the true purpose of the experiments to be hidden, unlike earlier approaches. The results of this study showed that participants rated stimuli such as cartoon strips as more funny when holding the pen with their teeth (which facilitated smiling) versus with their lips (which inhibited smiling).

The elegance of this approach led to this study being one of the most prominent and most cited in the literature regarding the FFH. However, recent efforts to replicate the original results of Strack and colleagues’ 1988 study have been unsuccessful in multiple labs (Wagenmakers et al., 2016), leading to a discussion regarding the true strength of the facial feedback effect (FFE).

In addition to the FFE, the posture of one’s entire body may also be a significant contributor to the perception of emotion. As Reed and McIntosh (2008) point out, the perception of body postures can be a crucial element of interpreting emotional states in others. It is therefore unsurprising that as with the FFE, the sensorimotoric feedback from one’s own body can induce emotional states (Duclos et al., 1989) and exert a significant influence on the perception of emotional stimuli such as emotional body postures (Wilbarger et al., 2011) and facial expressions. For example, faces appear to be more positive when evaluated during reaching motions in more comfortable postures, while uncomfortable reaching motions increased the accuracy of identifying facial expressions (Fantoni and Gerbino, 2014; Fantoni et al., 2014). In another example, participants seemed to vary in both self-reporting their own emotional states and assessing the motional states of others depending on whether they were placed in an upright expansive posture, which produced more positive reporting, as compared to a more slumped posture (Riskind and Gotay, 1982).

Our present study was motivated by a desire to contribute to the discussion concerning the effects of embodiment on emotion perception, as well as to extend this line to questioning into the domain of development, i.e., whether the strength of the embodiment effects might change over the course of development from childhood into adulthood. This possibility has been previously suggested based on the argument that until children gain sufficient experience and expertise in physically interacting with the surrounding world, effects of embodiment could impact their cognitive processes to a greater degree than might be the case for adults (Needham and Libertus, 2011). Furthermore, it is also possible that children might be more strongly influenced than adults by effects of embodiment on the processing of emotions because sensorimotoric feedback could play an anchoring role in the building of cognitive frameworks for processing abstract concepts such as emotions (Mandler, 2008; Winkielman et al., 2015).

We additionally decided to investigate the potential effects facial feedback could have on the processing of emotion words, as such stimuli are often encountered together with emotional faces during social interactions, and their perception may therefore be similarly affected by facial feedback. Evidence in support of this possibility was shown using a manipulation very similar to Strack’s original “pen-in-the-mouth,” which resulted in lower accuracy in judging words as being related to emotion depending on how the pen was held by participants (Niedenthal et al., 2009). More recently, in a study where participants evaluated words as positive, negative, or neutral, the participants showed selective facial muscle activity particularly during the perception of positive word stimuli (Weis and Herbert, 2017).

With the above goals in mind, we conducted a series of basic valence-based emotional categorization experiments using both facial expressions (Experiments 1a and 1b) and emotion words (Experiment 2) as targets. Child and adult participants were asked to categorize the target stimuli as positive or negative as quickly as possible. We believed that the simplicity of this task, originally adapted from one used by Tottenham et al. (2013), would not only allow for a fair comparison between children and adults but also make it suitable for testing a variety of manipulations intended to produce embodiment effects.

Using this task, we conducted our experiments using a manipulation involving the facial musculature based on the original method used by Strack et al. (1988) for the categorization of emotional words and faces, as well as a task that manipulated body posture in order to test whether a crouched or upright posture would influence the categorization of emotional facial expressions.

## Experiments 1a and 1b

Experiments 1a and 1b tested for effects of embodiment on the categorization of facial expressions.

In Experiment 1a, we used a manipulation of the facial musculature based on the classic “pen-in-the-mouth” task (Strack et al., 1988). Participants held a straw^1^ either with their teeth in the positive condition or their lips in the negative condition (in separate blocks). This approach was designed to either facilitate or inhibit the musculature involved in smiling, respectively.

In Experiment 1b, we used a manipulation of back posture in an attempt test for a broader range of possible embodiment effects on the categorization of faces. In the positive condition, participants either held a ball with their back producing an upright posture to facilitate the processing of positive stimuli while performing the categorization. In the negative condition, participants held a ball with their stomach producing a hunched posture to facilitate the processing of negative stimuli while performing the categorization.

To test for developmental differences in effects of embodiment in Experiments 1a and 1b, we tested both 9-year-old children and adults. The age of 9 was chosen for the children’s group because it was the midpoint of the age range of 6–12 years in which two previous studies (Vesker et al., 2018a,b) found significant differences in terms of the processing of positive and negative faces using the same facial expression categorization task used in Experiments 1a and 1b.

### Methods

#### Participants

Twenty 9-year-old children (9 females, 11 males) and 20 adult students (15 females, 5 males; mean age: 23 years) were recruited and tested at the University of Giessen in Germany. All participants were native German speakers, and the children were verified to be typically developing using the Raven’s Progressive Colored Matrices (Raven, 1974) non-verbal intelligence test, as well as the WWT 6–10 (“Wortschatz- und Wortfindungstest für 6– bis 10–Jährige“) German vocabulary test (Glück, 2011).

#### Stimuli

Stimuli consisted of 48 (24 positive, 24 negative) colored photographs of facial expressions by eight models (four men, four women), each of whom contributed three positive and three negative expressions. All photographs were provided by the laboratory of Marc D. Pell at McGill University (Pell, 2005). The positive and negative stimulus categories were balanced in terms of arousal and valence (Vesker et al., 2017).

#### Procedure

Participants were informed about the testing procedure and signed informed consent forms (by parents in the case of the child participants). Each participant participated in both the face-embodiment (Experiment 1a) and the posture-embodiment (Experiment 1b) experiments, in an order that was counterbalanced across participants within each age group.

Both Experiments 1a and 1b involved participants categorizing facial stimuli as positive or negative as quickly as possible. Each experiment contained two blocks (in a randomized counterbalanced order) during which participants adopted an embodiment state congruent with either positive or negative emotional states, and by extension the stimuli to be categorized. During each block, participants categorized all 48 faces, for a total of 96 trials per experiment. OpenSesame version 2.9 (Mathôt et al., 2012) was used as the controlling software to present stimuli and collect participant responses.

Positive embodiment states consisted of holding a straw with the teeth, approximating a smile (Experiment 1a), and holding a volleyball between the upper back and the back of the chair, producing an upright posture (Experiment 1b).

Negative embodiment states consisted of holding a straw with the lips, inhibiting smiling (Experiment 1a), and holding a volleyball between the stomach and the thighs, producing a hunched-over posture (Experiment 1b).

### Results

#### Analysis

For our analysis, we only considered data from participants who achieved an accuracy rate of at least 60% overall (all participants achieved this criterion). In addition, the data of individual trials were excluded if the response time for these trials exceeded the limit of 2 SDs over the mean response time for each age group. This criterion led to the exclusion of 358 trials from Experiment 1a, leaving 3482 trials to be analyzed, and 281 trials from Experiment 1b, leaving 3559 trials to be analyzed.

For each experiment, we carried out two ANOVAs in SPSS version 22 using the raw per-trial measures of accuracy and the response time for correctly responded trials as the dependent variables in their respective analyses. The valence of the target face (valence), the participant’s age (age), and the congruency of the embodiment condition and target face (congruency) served as the independent variables.

#### Experiment 1a (Straw)

##### Accuracy

The analysis of accuracy revealed a main effect of age [*F*(1,3474) = 10.524, *p* = 0.001, partial η*^2^* = 0.003], with adults showing a higher average accuracy than the children (97.3% versus 95.2%, respectively). We also found a main effect of valence [*F*(1,3474) = 12.148, *p* < 0.001, partial η*^2^* = 0.003], with negative faces being on average categorized more accurately than positive faces (97.3% versus 95.1%, respectively). No significant main effect or interactions of congruency of the embodiment condition were found (see Table 1).

**TABLE 1:** Face categorization under facial musculature and posture manipulation.

**Experiment**	**Participant** **age group**	**Measurement**	**Congruent** **condition**	**Incongruent** **condition**	**Difference** **(Cong. – Incong.)**
1a (straw)	Adults	Accuracy	97.7%	96.8%	0.9%^ns^
1a (straw)	Children	Accuracy	95.5%	94.8%	0.7%^ns^
1a (straw)	Adults	Response time	785 ms	798 ms	−13 ms^ns^
1a (straw)	Children	Response time	1121 ms	1115 ms	7 ms^ns^
1b (ball)	Adults	Accuracy	97.9%	97.6%	0.3%^ns^
1b (ball)	Children	Accuracy	97.0%	96.3%	0.7%^ns^
1b (ball)	Adults	Response time	790 ms	801 ms	−11 ms^ns^
1b (ball)	Children	Response time	1113 ms	1109 ms	4 ms^ns^

*Average values of accuracy and response time for correct trials in the congruent and incongruent conditions by children and adults in Experiments 1a and 1b. ns, indicates that the difference between the average values in the congruent and incongruent conditions did not reach statistical significance.*

##### Response Time

The analysis of response time of correct trials revealed a main effect of age [*F*(1,3342) = 845.827, *p* < 0.001, partial η*^2^* = 0.202], with adults showing a faster average response time than the children (791 ms versus 1119 ms, respectively). We also found a main effect of valence [*F*(1,3342) = 7.739, *p* = 0.005, partial η*^2^* = 0.002], with positive faces being on average categorized faster than negative faces (939 ms versus 971 ms, respectively). No significant main effect or interactions of congruency of the embodiment condition were found (see Table 1).

#### Experiment 1b (Ball)

##### Accuracy

The analysis of accuracy revealed a main effect of age [*F*(1,3551) = 4.147, *p* = 0.042, partial η*^2^* = 0.001], with adults showing a higher average accuracy than the children (97.8% versus 96.6%, respectively). We also found a main effect of valence [*F*(1,3551) = 6.710, *p* = 0.010, partial η*^2^* = 0.002], with negative faces being on average categorized more accurately than positive faces (97.9% versus 96.5%, respectively). No significant main effect or interactions of congruency of the embodiment condition were found (see Table 1).

##### Response Time

The analysis of response time of correct trials revealed a main effect of age [*F*(1,3451) = 846.492, *p* < 0.001, partial η*^2^* = 0.197], with adults showing a faster average response time than the children (795 ms versus 1111 ms, respectively). We also found a main effect of valence [*F*(1,3451) = 19.096, *p* < 0.001, partial η*^2^* = 0.006], with positive faces being on average categorized faster than negative faces (930 ms versus 977 ms, respectively). No significant main effect or interactions of congruency of the embodiment condition were found (see Table 1).

## Experiment 2

In Experiment 2, we looked for effects of embodiment on the categorization of emotion words using a similar task, since a previous study has shown developmental changes in the perception of positive versus negative words using such a task, albeit only in children up to the age of 6 years (Bahn et al., 2017b).

To test whether any facial effects of embodiment changed in the course of development, we tested 6-year-old children and adults in Experiment 2 using the same straw-based face manipulation as that in Experiment 1a. The age of 6 was chosen for the children’s group because it was the oldest age at which a previous study (Bahn et al., 2017b) was able to find significant effects using the same emotion word categorization task used in this experiment.

### Methods

#### Participants

Twenty-two 6-year-old children (14 females, 8 males) were recruited and tested at the University of Marburg. In addition, 20 adult students (16 females, 4 males; mean age: 22 years) were recruited and tested with the same task at the University of Giessen in Germany.

All participants grew up as monolingual native German speakers, and typical development for the children was verified in the same manner as in Experiment 1.

#### Stimuli

The stimulus material in the categorization task consisted of 48 German emotion terms, which were previously used in several other studies [Bahn et al., 2017a; Vesker et al., 2018b; see Bahn et al. (2017a) for a detailed description of the stimulus construction]. Twenty-four words had a positive valence (such as “hoffen,” engl. “to hope”) and 24 were negative (e.g., “leiden” engl. “to suffer”). These two sets of words, including nouns, verbs, and adjectives, were selected from the Berlin Affective Word List, which is a corpus of approximately 3000 German words (emotion terms as well as affective words and neutral words) obtained by Võ et al. (2009). The word sets were controlled for the variables of valence and arousal using values of previously collected valence and arousal ratings from 60 typically developed 9-year-old children (Bahn et al., 2017a). In addition, several linguistic variables were controlled that are known to influence word processing. For the final word sets, it was confirmed that:

(1) positive and negative emotion terms significantly differ in their mean value of valence, with positive words being clearly more positive than negative words;
(2) both word sets did not significantly differ in their mean value of arousal; and
(3) the mean values for frequency, concreteness, word class, word length, age of acquisition, neighborhood density, and the acoustic parameters of spoken word length (mean duration) and pitch showed no significant differences between positive and negative stimuli.

All words were recorded in a soundproofed booth, spoken by one female and one male trained native speakers of standard German using neutral prosody.

#### Procedure

The testing procedure was analogous to that used in Experiment 1a, but with the participants tasked with categorizing emotion words instead of facial expressions as positive or negative.

Each of the 48 emotion terms was presented via headphones twice, once congruently primed and once incongruently primed by one of the two embodiment states previously used in Experiment 1a (holding the straw with the teeth for a positive state and with the lips for a negative state) in separate blocks for a total of 96 trials.

### Results

#### Analysis

The collected data were prepared for analysis using the same criteria as in Experiments 1a and 1b, with only those participants who achieved an overall accuracy of at least 60% being included in our analysis, leading to the exclusion of 6 out of the 22 tested 6-year-old children. Four hundred twenty-four trials where the participant’s response time was above 2 SDs over the mean of the age group’s mean response time were also excluded from analysis, leaving a total of 3312 trials to be analyzed.

Again, ANOVAs were carried out, one using the raw per-trial accuracy for each participant as the dependent variable and the other using raw per-trial response times for correctly responded trials as the dependent variable. Independent variables were the valence of the target words (valence), the participant’s age group (age), and the congruency between the embodiment state and target valence (congruency).

#### Accuracy

The analysis of accuracy revealed a main effect of age [*F*(1,3304) = 161.946, *p* < 0.001, partial η*^2^* = 0.047], with adults showing a higher average accuracy than the children (96.9% versus 84.8%, respectively). We also found a main effect of valence [*F*(1,3304) = 41.794, *p* < 0.001, partial η*^2^* = 0.012]: positive words were on average categorized more accurately than negative words (93.9% versus 87.8%, respectively). A significant interaction between age and valence was also detected [*F*(1,3304) = 10.495, *p* = 0.001, partial η*^2^* = 0.003] with the gap between the accuracy of categorizing positive and negative words being larger in magnitude in children compared to adults. We found no significant main effect of the congruency of the embodiment condition or interactions between this factor and any of the other factors (see Table 2).

**TABLE 2:** Word categorization under facial musculature manipulation.

**Participant** **age group**	**Measurement**	**Congruent** **condition**	**Incongruent** **condition**	**Difference** **(Cong. – Incong.)**
Adults	Accuracy	97.4%	96.3%	1.1%^ns^
Children	Accuracy	83.8%	85.9%	−2.1%^ns^
Adults	Response time	374 ms	374 ms	0 ms^ns^
Children	Response time	1262 ms	1270 ms	−8 ms^ns^

*Average values of accuracy and response time for correct trials in the congruent and incongruent conditions by children and adults. ns, indicates that the difference between the average values in the congruent and incongruent conditions did not reach statistical significance.*

#### Response Time

The analysis of response time of correct trials revealed a main effect of age [*F*(1,3024) = 1542.497, *p* < 0.001, partial η*^2^* = 0.338], with adults showing a faster average response time than the children (372 ms versus 1242 ms, respectively). We also found a main effect of valence [*F*(1,3304) = 13.502, *p* < 0.001, partial η*^2^* = 0.004]: Positive words were on average categorized faster than negative words (766 ms versus 847 ms, respectively). A significant interaction between age and valence was also detected [*F*(1,3304) = 15.520, *p* < 0.001, partial η*^2^* = 0.005], with the gap between the speed of categorizing positive and negative words being individually significant in children but not adults. We found no significant main effect of the congruency of the embodiment condition or interactions between this factor and any of the other factors (see Table 2).

## Discussion

The results of our experiments reproduced the early positivity bias for words and faces reported by earlier studies using the same valence-based emotional categorization task (Bahn et al., 2017b; Vesker et al., 2018a), wherein younger children tended to show faster and more accurate responses to positive stimuli versus negative stimuli, with this positive/negative disparity diminishing with increasing age.

However, we did not detect any significant effects of embodiment on the categorization of either faces (Experiment 1a) or words (Experiment 2) using the manipulation of facial musculature to either facilitate or inhibit smiling based on the original “pen-in-the-mouth” approach described by Strack et al. (1988). Having not been able to detect embodiment effects using the face manipulation, we attempted to detect such effects using different manipulations.

We manipulated posture by asking participants to either hold a ball with their back against the chair, producing an upright posture, or between their stomach and legs, producing a hunched-over posture as they performed our face-categorization task (Experiment 1b). However, despite body manipulations having been shown to affect the perception of facial emotions (Fantoni and Gerbino, 2014; Fantoni et al., 2014) and emotions in general (Riskind and Gotay, 1982), we did not find a significant effect of the posture manipulation on the categorization of faces.

In summary, using our methodology, we were unable to detect any significant effects of embodiment on the categorization of either facial expressions or emotion words in either children or adults. Our sample sizes were similar to those used in a study using a nearly identical paradigm (albeit with word primes instead of embodiment manipulations), which showed a significant main effect of priming congruency, as well as significant interactions involving priming congruency (Vesker et al., 2018b). This suggests that either embodiment does not affect the categorization process of emotional stimuli, or that our methodology was simply not sensitive enough to detect such effects given our sample size. The latter notion is supported by argument made by Fritz Strack himself in response to the overwhelming failure of other researchers to replicate the “pen-in-the-mouth” effect of embodiment (Wagenmakers et al., 2016). Strack (2016) argued that a crucial detail of the replication efforts, that participants were recorded to ensure they followed the procedure accurately, may have interfered with the effect of embodiment. By contrast, in the original study by Strack et al. (1988), subjects were entirely unobserved. This suggestion that the participants’ knowledge of being observed might have a disrupting influence on the FFE has recently received some support from a study that indeed showed a stronger FFE when participants were not recorded or observed (Noah et al., 2018). This effect of observation might thus have also contributed to our inability to detect significant embodiment effects in the present study, as our participants were under the experimenter’s observation during data collection. However, it is not entirely clear whether this effect of observation can be eliminated while also ensuring that participants, particularly children, properly follow the experimental procedure. Thus, in addition to testing larger samples of participants, future studies investigating such effects from a developmental perspective would most likely require some degree of covert observation of participants in order to satisfy the above requirements.

It must also be pointed out that the materials used in our experiments, including both the stimuli and the straws, differed from the materials used in the original method of Strack et al. (1988), as well as recent successful replications of the FFE by Noah et al. (2018) and Marsh et al. (2018). Unlike our study, these studies used cartoon strips as stimuli to be rated, and pens or pencils for the embodiment manipulation, which are more rigid than the straws in our experiment. These differences in materials could have played a role in our results, as a recent meta-analysis of the FFE by Coles et al. (2019) found that the effect tended to be relatively small in magnitude and sensitive to the types of stimuli used. Therefore, it is also important to acknowledge that with regard to Experiments 1a and 2, all the deviations from the classic “pen-in-the-mouth” task mentioned above may have interfered with the FFE to a degree which could have made it more difficult to detect.

A final point to consider regarding Experiments 1a and 2 is that the FFA could have been reduced in magnitude by the very nature of our task. By asking participants to merely categorize stimuli as positive or negative, we avoided the need for them to retrieve specific emotion labels in order to make the comparison between the positive and negative categories more balanced (Kauschke et al., 2019). However, it is possible that because the muscles manipulated with the straws are linked to the specific expressions involving the mouth, this manipulation may not have had a significant impact on the type of higher-level categorization that our participants performed. If true, our results would suggest that the FFE might apply more to specific emotional states rather than to the perception of broader valence categories, and that this relationship does not vary between childhood and adulthood.

The present study was conducted in accordance with the German Psychological Society Research Ethics Guidelines. The Office of Research Ethics at the University of Giessen approved the experimental procedure and the informed consent protocol. Written informed consent was obtained from adult participants and the parents of child participants prior to their participation in the study.

## Data Availability Statement

The datasets used for our analyses are available on request to the corresponding author, as well as on the Zenodo platform under the title of this article.

## Ethics Statement

The studies involving human participants were reviewed and approved by The Office of Research Ethics at the University of Giessen. Written informed consent to participate in this study was provided by the adult participants, and by the legal guardian/next of kin of the child participants.

## Author Contributions

MV and DB involved in the study design, data collection, analysis, and production of the manuscript. CK and GS involved in the study design, analysis, and production of the manuscript. MN and CS involved in the study design, data collection, and analysis.

## Conflict of Interest

The authors declare that the research was conducted in the absence of any commercial or financial relationships that could be construed as a potential conflict of interest.

## Footnotes

1 Individually packaged straws were substituted for the pen used in the original “pen-in-the-mouth” task for hygienic purposes.

## References

[B1] Bahn D. Kauschke C. Vesker M. Schwarzer G. (2017a). Perception of valence and arousal in German emotion terms: a comparison between 9-year-old children and adults. *Appl. Psycholinguist.* 39 463–481. 10.1017/S0142716417000443

[B2] Bahn D. Vesker M. García Alanis J. C. Schwarzer G. Kauschke C. (2017b). Age-dependent positivity-bias in children’s processing of emotion terms. *Front. Psychol.* 8:1268. 10.3389/fpsyg.2017.01268 28798706 PMC5526962

[B3] Buck R. (1980). Nonverbal behavior and the theory of emotion: the facial feedback hypothesis. *J. Pers. Soc. Psychol.* 38 811–824. 10.1037/0022-3514.38.5.811 7381683

[B4] Coles N. A. Larsen J. T. Lench H. C. (2019). A meta-analysis of the facial feedback literature: effects of facial feedback on emotional experience are small and variable. *Psychol. Bull.* 145 610–651. 10.1037/bul0000194 30973236

[B5] Darwin C. (1872). *The Expression of the Emotions in Man and Animals.* London: John Marry.

[B6] Duclos S. E. Laird J. D. Schneider E. Sexter M. Stern L. Van Lighten O. (1989). Emotion-specific effects of facial expressions and postures on emotional experience. *J. Pers. Soc. Psychol.* 57 100–108. 10.1037/0022-3514.57.1.100

[B7] Fantoni C. Cavallero C. Gerbino W. (2014). “The motor action mood induction procedure affects the detection of facial emotions,” in *Proceedings of the Trieste Symposium on Perception and Cognition, November 27th-28th 2014*, Rovereto, 79–81.

[B8] Fantoni C. Gerbino W. (2014). Body actions change the appearance of facial expressions. *PLoS One* 9:e0108211. 10.1371/journal.pone.0108211 25251882 PMC4176726

[B9] Glück C. W. (2011). *Wortschatz- und Wortfindungstest für 6– bis 10–Jährige: WWT 6–10.* Munich: Elsevier.

[B10] Kauschke C. Bahn D. Vesker M. Schwarzer G. (2019). The role of emotional valence for the processing of facial and verbal stimuli—positivity or negativity bias? *Front. Psychol.* 10:e01654. 10.3389/fpsyg.2019.01654 31402884 PMC6676801

[B11] Laird J. D. (1984). The real role of facial response in the experience of emotion: a reply to tourangeau and ellsworth, and others. *J. Pers. Soc. Psychol.* 47 909–917. 10.1037//0022-3514.47.4.909 501520

[B12] Mandler J. M. (2008). On the birth and growth of concepts. *Philos. Psychol.* 21 207–230. 10.1080/09515080801980179

[B13] Marsh A. A. Rhoads S. A. Ryan R. M. (2018). A multi-semester classroom demonstration yields evidence in support of the facial feedback effect. *Emotion* 19 1500–1504. 10.1037/emo0000532 30475036

[B14] Mathôt S. Schreij D. Theeuwes J. (2012). OpenSesame: an open-source, graphical experiment builder for the social sciences. *Behav. Res. Methods* 44 314–324. 10.3758/s13428-011-0168-7 22083660 PMC3356517

[B15] Needham A. Libertus K. (2011). Embodiment in early development. *Cogn. Sci.* 2 117–123. 10.1002/wcs.109 26301917

[B16] Niedenthal P. M. Barsalou L. W. Winkielman P. Krauth-gruber S. Ric F. (2005). Embodiment in attitudes, social perception, and emotion. *Pers. Soc. Psychol. Rev.* 9 184–211. 10.1207/s15327957pspr0903 16083360

[B17] Niedenthal P. M. Winkielman P. Mondillon L. Vermeulen N. (2009). Embodiment of emotion concepts. *J. Pers. Soc. Psychol.* 96 1120–1136. 10.1037/a0015574 19469591

[B18] Noah T. Schul Y. Mayo R. (2018). When both the original study and its failed replication are correct: feeling observed eliminates the facial-feedback effect. *J. Pers. Soc. Psychol.* 114 657–664. 10.1037/pspa0000121 29672101

[B19] Pell M. D. (2005). Nonverbal emotion priming: evidence from the “facial affect decision task.”. *J. Nonverb. Behav.* 29 45–73. 10.1007/s10919-004-0889-8

[B20] Raven J. C. (1974). *Coloured Progressive Matrices.* San Antonio, TX: Pearson.

[B21] Reed C. L. McIntosh D. N. (2008). “The social dance: on-line body perception in the context of others,” in *Embodiment, Ego-Space, and Action*, 1st Edn, eds Klatzky R. L. MacWhinney B. Behrmann M. (Abingdon: Psychology Press).

[B22] Riskind J. H. Gotay C. C. (1982). Physical posture: could it have regulatory or feedback effects on motivation and emotion? *Motivat. Emot.* 6 273–298. 10.1007/bf00992249

[B23] Strack F. (2016). Reflection on the smiling registered replication report. *Perspect. Psychol. Sci.* 11 929–930. 10.1177/1745691616674460 27784750

[B24] Strack F. Martin L. L. Stepper S. (1988). Inhibiting and facilitating conditions of the human smile: a nonobtrusive test of the facial feedback hypothesis. *J. Pers. Soc. Psychol.* 54 768–777. 10.1037//0022-3514.54.5.768 3379579

[B25] Tomkins S. S. (1962). “Chapter 7 the primary site of the affects: the face,” in *Affect Imagery Consciousness*, ed. Karon B. P. (Berlin: Springer), 113–134.

[B26] Tottenham N. Phuong J. Flannery J. Gabard-Durnam L. Goff B. (2013). A negativity bias for ambiguous facial-expression valence during childhood: converging evidence from behavior and facial corrugator muscle responses. *Emotion* 13 92–103. 10.1037/a0029431 22906084 PMC4006094

[B27] Vesker M. Bahn D. Degé F. Kauschke C. Schwarzer G. (2017). Perceiving arousal and valence in facial expressions: differences between children and adults. *Eur. J. Dev. Psychol.* 5629 1–15. 10.1080/17405629.2017.1287073

[B28] Vesker M. Bahn D. Dege F. Kauschke C. Schwarzer G. (2018a). Developmental changes in the categorical processing of positive and negative facial expressions. *PLoS One* 13:e0201521. 10.1371/journal.pone.0201521 30075000 PMC6075754

[B29] Vesker M. Bahn D. Kauschke C. Tschense M. Degé F. Schwarzer G. (2018b). Auditory emotion word primes influence emotional face categorization in children and adults, but not vice versa. *Front. Psychol.* 9:1–10. 10.3389/fpsyg.2018.00618 29765346 PMC5938388

[B30] Võ M. L. H. Conrad M. Kuchinke L. Urton K. Hofmann M. J. Jacobs A. M. (2009). The berlin affective word list reloaded (BAWL-R). *Behav. Res. Methods* 41 534–538. 10.3758/BRM.41.2.534 19363195

[B31] Wagenmakers E.-J. Beek T. Dijkhoff L. Gronau Q. F. Acosta A. Adams R. B. (2016). Registered replication report: strack, martin, & stepper (1988). *Perspect. Psychol. Sci.* 11 917–928. 10.1177/1745691616674458 27784749

[B32] Weis P. P. Herbert C. (2017). Bodily reactions to emotional words referring to own versus other people’s emotions. *Front. Psychol.* 8:1277. 10.3389/fpsyg.2017.01277 28878699 PMC5572286

[B33] Wilbarger J. L. Reed C. L. McIntosh D. N. (2011). Implicit influence of affective postures on the perception of others: you can’t show me how i feel. *Emotion* 11 481–491. 10.1037/a0023271 21668101

[B34] Winkielman P. Niedenthal P. Wielgosz J. Eelen J. Kavanagh L. C. (2015). “Embodiment of cognition and emotion,” in *APA Handbook of Personality and Social Psychology*, Vol. 1 eds Mikulincer M. Shaver P. R. (Washington, D.C: American Psychological Association), 151–175.

